# Dock3 interaction with a glutamate-receptor NR2D subunit protects neurons from excitotoxicity

**DOI:** 10.1186/1756-6606-6-22

**Published:** 2013-05-04

**Authors:** Ning Bai, Hideki Hayashi, Tomomi Aida, Kazuhiko Namekata, Takayuki Harada, Masayoshi Mishina, Kohichi Tanaka

**Affiliations:** 1 Laboratory of Molecular Neuroscience, Medical Research Institute, Tokyo Medical and Dental University, 1-5-45 Yushima, Bunkyo-ku, Tokyo 113-8510, Japan; 2 The Center for Brain Integration Research, Tokyo Medical and Dental University, Tokyo, Japan; 3 JST, CREST, Saitama, Japan; 4 College of Basic Medicine, China Medical University, 92 Bei Er Road, Heping District, Shenyang 110001, China; 5 Priority Organization for Innovation and Excellence, Kumamoto University, 509 General Science Building, Honjo 1-1-1, Kumamoto 860-8556, Japan; 6 Visual Research Project, Tokyo Metropolitan Institute of Medical Science, 2-1-6 Kamikitazawa, Setagaya-ku, Tokyo 156-8506, Japan; 7 Brain Science Laboratory, The Research Organization of Science and Technology, Ritsumeikan University, Nojihigashi 1-1-1, Kusatsu, Shiga 525-8577, Japan

**Keywords:** NMDA receptor, NR2D, Dock3, Excitotoxicity, Glaucoma, Memantine, Glutamate transporter

## Abstract

**Background:**

N-methyl-D-aspartate receptors (NMDARs) are critical for neuronal development and synaptic plasticity. Dysregulation of NMDARs is implicated in neuropsychiatric disorders. Native NMDARs are heteromultimeric protein complexes consisting of NR1 and NR2 subunits. NR2 subunits (NR2A–D) are the major determinants of the functional properties of NMDARs. Most research has focused on NR2A- and/or NR2B-containing receptors. A recent study demonstrated that NR2C- and/or NR2D-containing NMDARs are the primary targets of memantine, a drug that is widely prescribed to treat Alzheimer’s disease. Our laboratory demonstrated that memantine prevents the loss of retinal ganglion cells (RGCs) in GLAST glutamate transporter knockout mice, a model of normal tension glaucoma (NTG), suggesting that NR2D-containing receptors may be involved in RGC loss in NTG.

**Results:**

Here we demonstrate that NR2D deficiency attenuates RGC loss in GLAST-deficient mice. Furthermore, Dock3, a guanine nucleotide exchange factor, binds to the NR2D C-terminal domain and reduces the surface expression of NR2D, thereby protecting RGCs from excitotoxicity.

**Conclusions:**

These results suggest that NR2D is involved in the degeneration of RGCs induced by excitotoxicity, and that the interaction between NR2D and Dock3 may have a neuroprotective effect. These findings raise the possibility that NR2D and Dock3 might be potential therapeutic targets for treating neurodegenerative diseases such as Alzheimer’s disease and NTG.

## Background

Glutamate is the major excitatory neurotransmitter in the mammalian central nervous system (CNS) and plays an essential role in neural development and information processing through a variety of ionotropic (ligand-gated) and metabotropic (G-protein–coupled) receptors [1]. However, increased levels of glutamate results in extensive stimulation of these receptors, which can eventually become neurotoxic [2,3]. Overstimulation of N-methyl-D-aspartate receptors (NMDARs) is implicated in many diseases, including epilepsy, schizophrenia, and various neurodegenerative disorders [4-7].

Molecular cloning methods have identified multiple NMDAR subunits, including NR1, a family of NR2 subunits (NR2A–NR2D), and two NR3 subunits (NR3A and NR3B). Native NMDARs are heteromultimeric protein complexes composed of NR1 and NR2 subunits, and in some cases NR3 subunits. NR2 subunits are major determinants of the functional properties of NMDARs, including characteristics such as agonist affinity, deactivation kinetics, single-channel conductance, Ca^2+^ permeability, and sensitivity to Mg^2+^. Since NR2A- and NR2B-containing receptors are highly expressed in the cortex, and NR2C- and NR2D-containing receptors have low opening probabilities and low single-channel conductances, most previous research has focused on NR2A- and/or NR2B-containing receptors [8-10].

Recently, it was demonstrated that Mg^2+^ regulates the sensitivity of NMDARs to memantine, a drug that is widely prescribed for the treatment of Alzheimer’s disease [11]. In a physiological concentration (1 mM) of extracellular Mg^2+^, memantine exerts a more potent blocking effect at NR2C/D subunits than NR2A/B subunits. These findings suggest that NR2C- and/or NR2D-containing NMDARs are likely to be the main targets of memantine. In comparison with the NR2C subunit, NR2D is a particularly interesting NMDAR subunit because it mediates the mechanisms by which phencyclidine (PCP) induces locomotor hyperactivity in a novel environment, behaviors thought to model positive symptoms of schizophrenia [12]. NR2D subunits are broadly expressed in the adult mammalian brain, including the hippocampus, cortex and retina, all of which are regions of the CNS thought to be involved in Alzheimer’s disease, schizophrenia, and glaucoma [8,13-15]. Previously, our laboratory demonstrated that memantine prevented the loss of retinal ganglion cells (RGCs) in glutamate aspartate transporter (GLAST) knockout mice, a model of NTG [16], suggesting that NR2D-containing receptors may be involved in RGC loss in NTG. In the present study, we first investigated whether NR2D is involved in the excitotoxic degeneration of RGCs. Using yeast two-hybrid screening we identified NR2D-interacting molecules that modulate the function or localization of NR2D-containing NMDARs.

Here, we report that NR2D deficiency protects RGCs from excitotoxicity. In addition, we identified dedicator of cytokinesis 3 (Dock3) as a novel NR2D subunit interacting protein, and showed that the interaction between NR2D and Dock3 protects RGCs from excitotoxicity by reducing the surface expression of NR2D.

## Results

### NR2D deficiency prevents RGC death in GLAST-deficient mice

**Figure 1:**
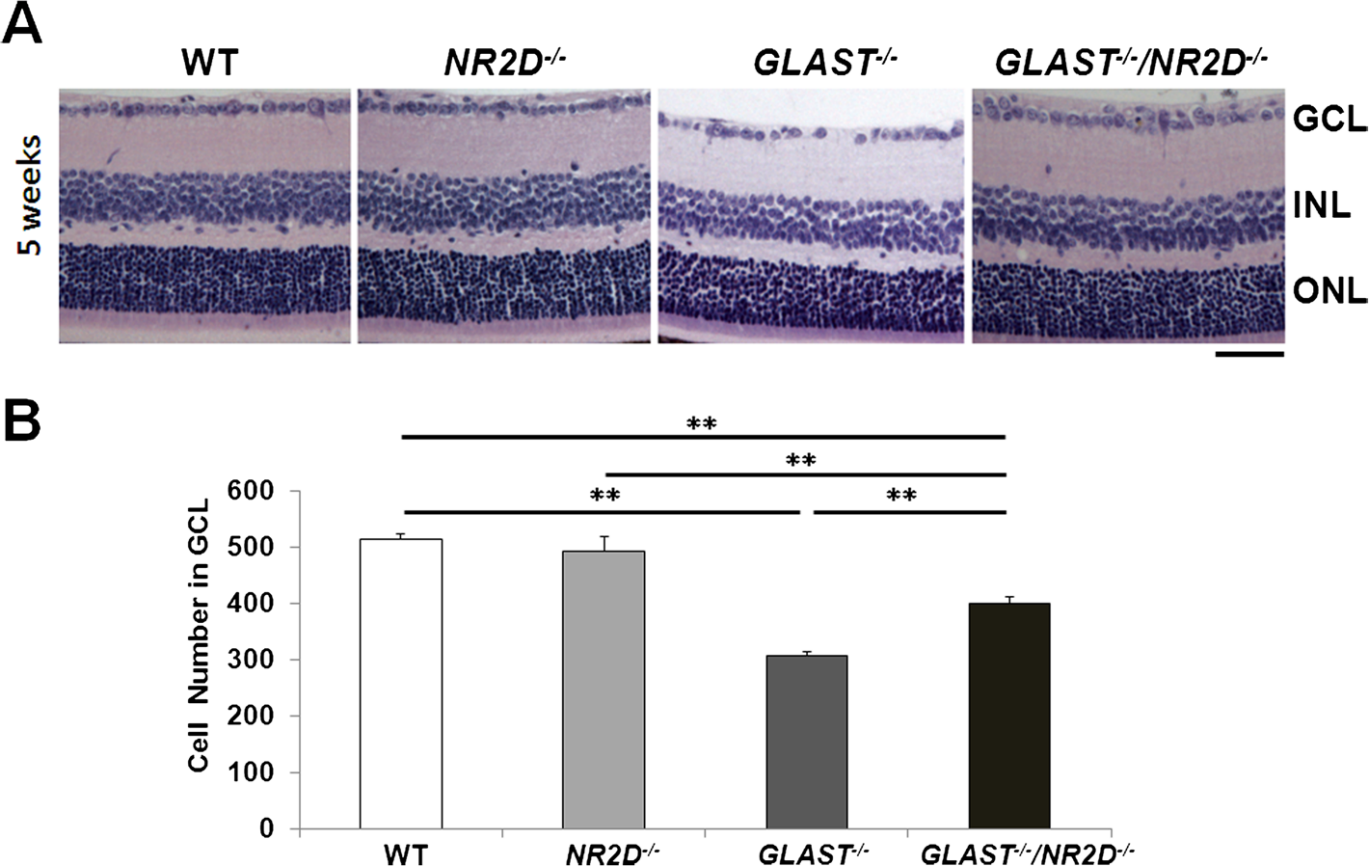
**NR2D deficiency prevents the loss of RGCs in GLAST-deficient mice.** (**A**) Hematoxylin and eosin staining of retinal sections at 5 weeks. Scale bar: 50 μm. (**B**) Quantification of RGC number in WT, *NR2D*^*−/−*^, *GLAST*^*−/−*^, and *GLAST*^*−/−*^/*NR2D*^*−/−*^ mice. The number of neurons in the GCL was counted in retinal sections from one ora serrata through the optic nerve to the other ora serrata. n = 5 per group. **p < 0.01.

To determine whether NR2D is involved in RGC degeneration in GLAST-deficient mice, we examined the histopathology of retinas from *GLAST*^*−/−*^ and *NR2D*^*−/−*^ mice. As shown in Figure 1, the retinas of *NR2D*^*−/−*^ mice showed normal organization at 5 weeks. The cell number in the ganglion cell layer (GCL) of *NR2D*^*−/−*^ mice (494 ± 26) was not significantly different from that in wild-type (WT) mice (514 ± 9), whereas the cell number in the GCL of *GLAST*^*−/−*^ mice was significantly less (307 ± 6) than that in WT. However, in *GLAST*^*−/−*^*NR2D*^*−/−*^ double-knockout mice, the number of GCL cells was significantly higher (401 ± 10) than that in *GLAST*^*−/−*^ mice, although it was still lower than that in WT and *NR2D*^*−/−*^ mice. These results suggest that NR2D deficiency protects against the loss of RGCs in GLAST-deficient mice.

### NR2D deficiency prevents NMDA-induced-excitotoxic retinal cell death

**Figure 2:**
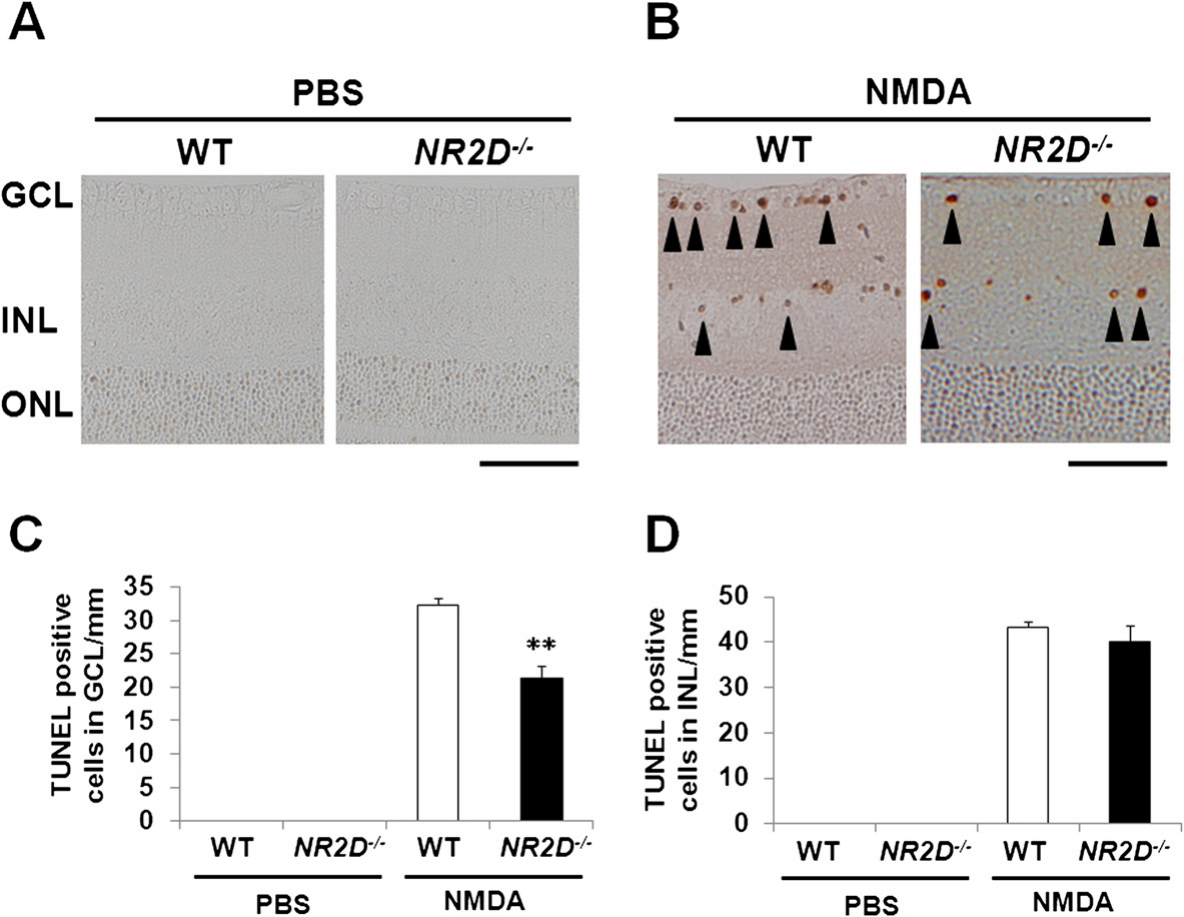
**NR2D deficiency prevents NMDA-induced-excitotoxic retinal cell death.** (**A** and **B**) TUNEL analysis of retinas of WT and *NR2D*^*−/−*^ mice 24 hr after PBS and NMDA injection. Arrowheads in **B** indicate TUNEL-positive cells. Scale bar: 50 μm. (**C** and **D**) The number of TUNEL-positive cells in the GCL (**C**) and INL (**D**). n = 5 per group. **p < 0.01.

In GLAST-deficient mice, both excitotoxicity and oxidative stress contribute to RGC degeneration [16]. To investigate whether NR2D deficiency reduces retinal cell death resulting from NMDA-induced excitotoxicity, we used TUNEL assay to examine the retinas of mice 24 h after an intravitreal injection of NMDA. No TUNEL-positive cells were detected in the controls or *NR2D*^*−/−*^ mice after injection of phosphate-buffered saline (PBS) (Figure 2A), whereas a number of TUNEL-positive cells were observed in the GCL and inner nuclear layer (INL) after injection of NMDA (Figure 2B). NR2D deficiency significantly reduced the mean number of TUNEL-positive cells in the GCL, but not in the INL (Figure 2C, D). These results suggest that NR2D deficiency protects against excitotoxicity-induced retinal cell death in the GCL.

### Identification of Dock3 as an NR2D-interacting molecule

**Figure 3:**
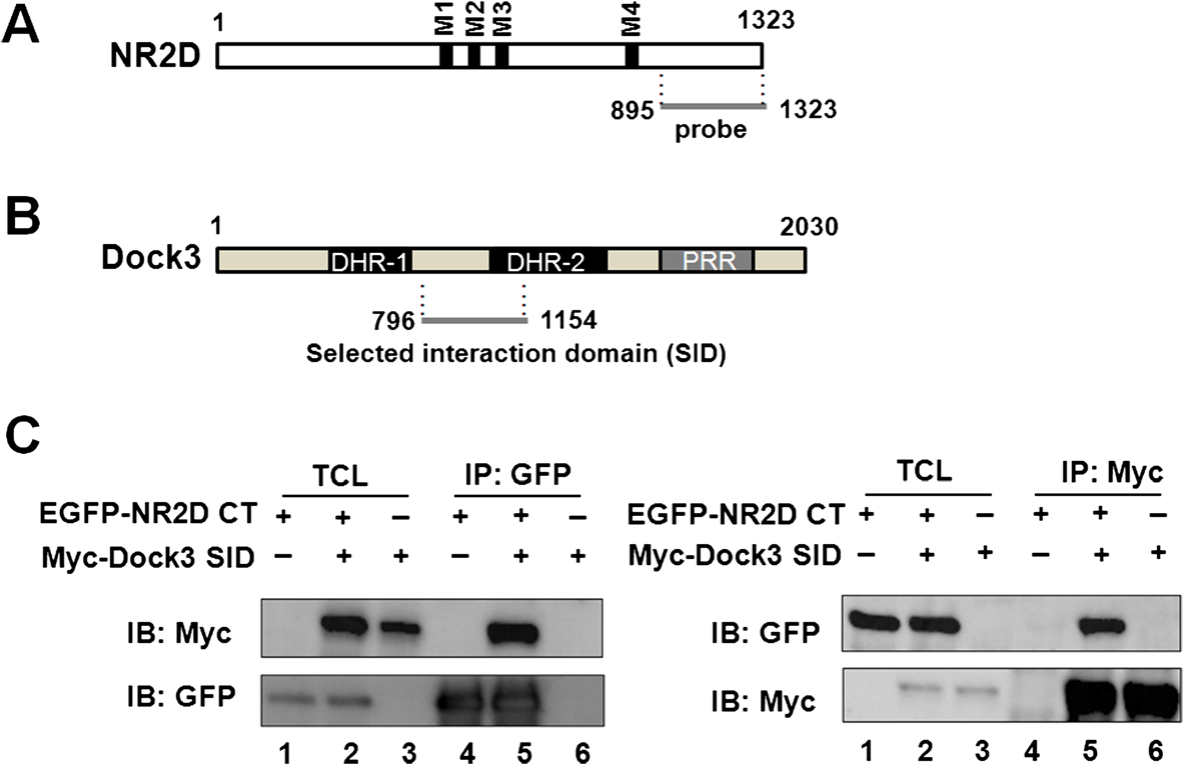
**Identification of Dock3 as an NR2D-interacting molecule.** (**A**) Schematic diagram of NR2D and a probe used for yeast two-hybrid screening. M1–M4 indicates the transmembrane regions. The numbers refer to the amino-acid positions of NR2D. (**B**) Schematic diagram of Dock3. The selected interaction domain (SID) corresponds to the minimal region common to all fragments identified by yeast two-hybrid screening. The numbers indicate the amino-acid positions within Dock3. DHR-1, Dock homology region 1; DHR-2, dock homology region 2; PRR, proline-rich region. (**C**) The interaction between NR2D and Dock3 in HEK293T cells. HEK293T cells were transfected with plasmids encoding the SID of Dock3 (Myc-Dock3 SID) and EGFP-tagged NR2D C-terminus (EGFP-NR2D CT). Lysates of transfected cells were immunoprecipitated (IP) with anti-GFP (left panel) or anti-Myc antibodies (right panel). Immune complexes were detected by Western blotting with anti-GFP or anti-Myc antibodies. In lanes 1–3, 1/10th volumes of the lysates used for immunoprecipitation were loaded for TCL samples. IB, Immunoblotting.

Multiple studies show that protein-protein interactions involving the intracellular C-terminal domains of NMDARs control the function and localization of these receptors [17-20]. To identify novel binding partners of the NR2D subunit, we performed a yeast two-hybrid screening of a mouse brain cDNA library using the C-terminal domain of NR2D (residues 895–1323) as bait (Figure 3A). Two of the 77 clones initially identified as interacting proteins encoded the Dock homology region 2 (DHR-2) domain (Figure 3B) of dedicator of cytokinesis 3 (Dock3), also known as modifier of cell adhesion (MOCA), which is specifically expressed in the CNS [21]. To investigate whether NR2D and Dock3 also interact in mammalian cells, human embryonic kidney (HEK) 293 T cells were transfected with expression plasmids encoding a Myc-tagged Dock3 interaction domain (amino acids 796–1154) and/or the NR2D C-terminus (amino acids 895–1323) carrying an EGFP tag. Protein lysates were prepared from the transfected cells for co-immunoprecipitation analyses. Western blots of anti-EGFP immunoprecipitates with an anti-Myc antibody revealed that the Dock3 interaction domain co-precipitated with the EGFP-tagged NR2D C-terminus only when both proteins were expressed (Figure 3C, left panel). Conversely, the NR2D C-terminus was present in anti-Myc immunoprecipitates of Dock3 interaction domain (Figure 3C, right panel), indicating that Dock3 was associated with the NR2D C-terminus in heterologous HEK293T cells.

**Figure 4:**
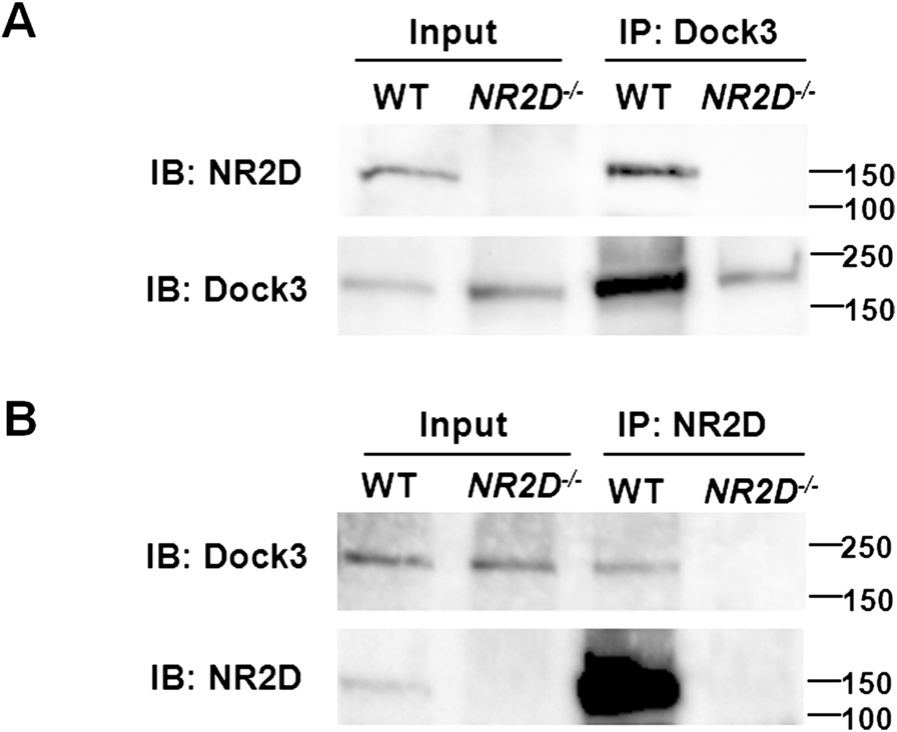
**Interaction of Dock3 with NR2D in brain.** (**A**) Identification of NR2D in Dock3 immunoprecipitates from WT, but not *NR2D*^*−/−*^ embryonic-brain homogenates. (**B**) Identification of Dock3 in NR2D immunoprecipitates from WT, but not *NR2D*^*−/−*^ embryonic-brain homogenates. The immunoprecipitates and brain lysates were subjected to immunoblotting with anti-NR2D and anti-Dock3 antibodies. IP, immunoprecipitation; IB, immunoblotting.

To determine whether Dock3 also interacted with NR2D in the retina, anti-NR2D immunoprecipitates from retina lysates were probed with an anti-Dock3 antibody. However, Dock3 was not detected in anti-NR2D immunoprecipitates, likely due to the low expression level of NR2D. Therefore, we performed co-immunoprecipitations using brain homogenates from mice at embryonic day 18, when the expression level of NR2D is high. The data clearly showed that Dock3 co-immunoprecipitated with NR2D (Figure 4A), and reciprocal co-immunoprecipitation experiments (Figure 4B) confirmed that Dock3 associated with NR2D in the embryonic brain.

Taken together, these results demonstrated that Dock3 effectively interacts with NR2D subunits in both cultured human cells and mouse embryonic brain.

### Dock3 and NR2D are co-expressed in the mouse retina

**Figure 5:**
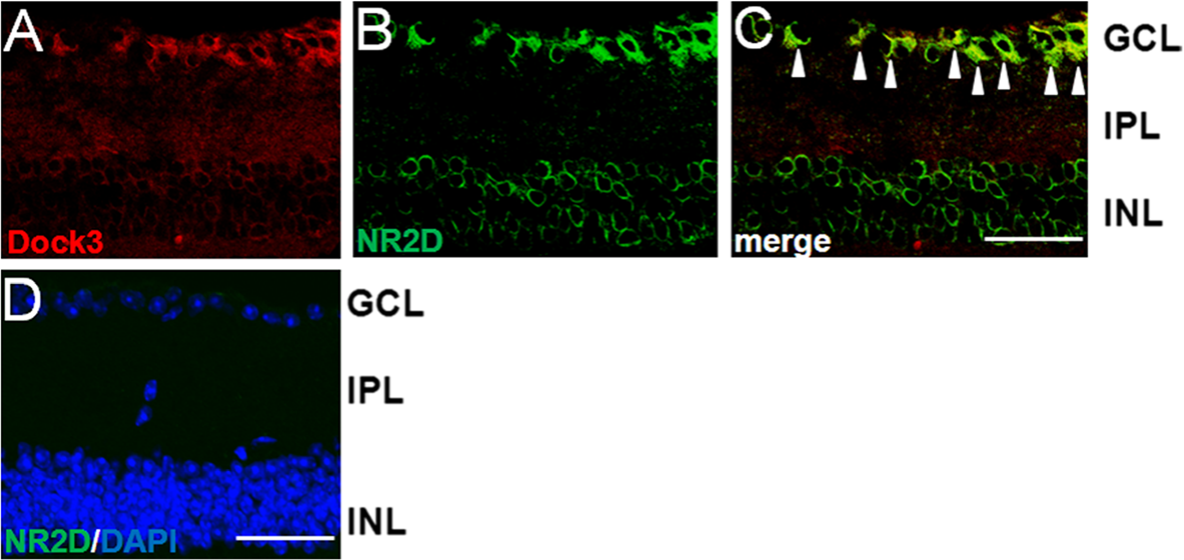
**Co-localization of NR2D and Dock3 in the mouse retina.** Double-labeling experiments were carried out on mouse retina sections (**A**-**C**). Dock3 (**A**) is red and NR2D (**B**) is green. Dock3 co-localized with NR2D in RGCs shown in the overlay panel (**C**) (arrowheads). Scale bar: 50 μm. (**D**) No NR2D immunoreactivity (green) was detected in retinas from *NR2D*^*−/−*^ mice. Nuclei are counterstained with DAPI (blue). Scale bar: 50 μm. IPL, inner plexiform layer.

Because NR2D is involved in excitotoxic degeneration of RGCs, we examined the distribution of Dock3 and NR2D expression in the mouse retina. Immunohistochemical analysis showed that Dock3 is expressed in the RGCs (Figure 5A). In addition, co-labeling with anti-NR2D antibodies revealed that Dock3 colocalizes with NR2D in these cells (Figure 5B, C). These data suggest that co-expression of NR2D and Dock3 occurs not only in the embryonic brain, but also in the GCL of the retina.

### Overexpression of Dock3 inhibits glutamate-induced intracellular Ca^2+^ elevation and prevents glutamate-induced apoptosis in RGCs

**Figure 6:**
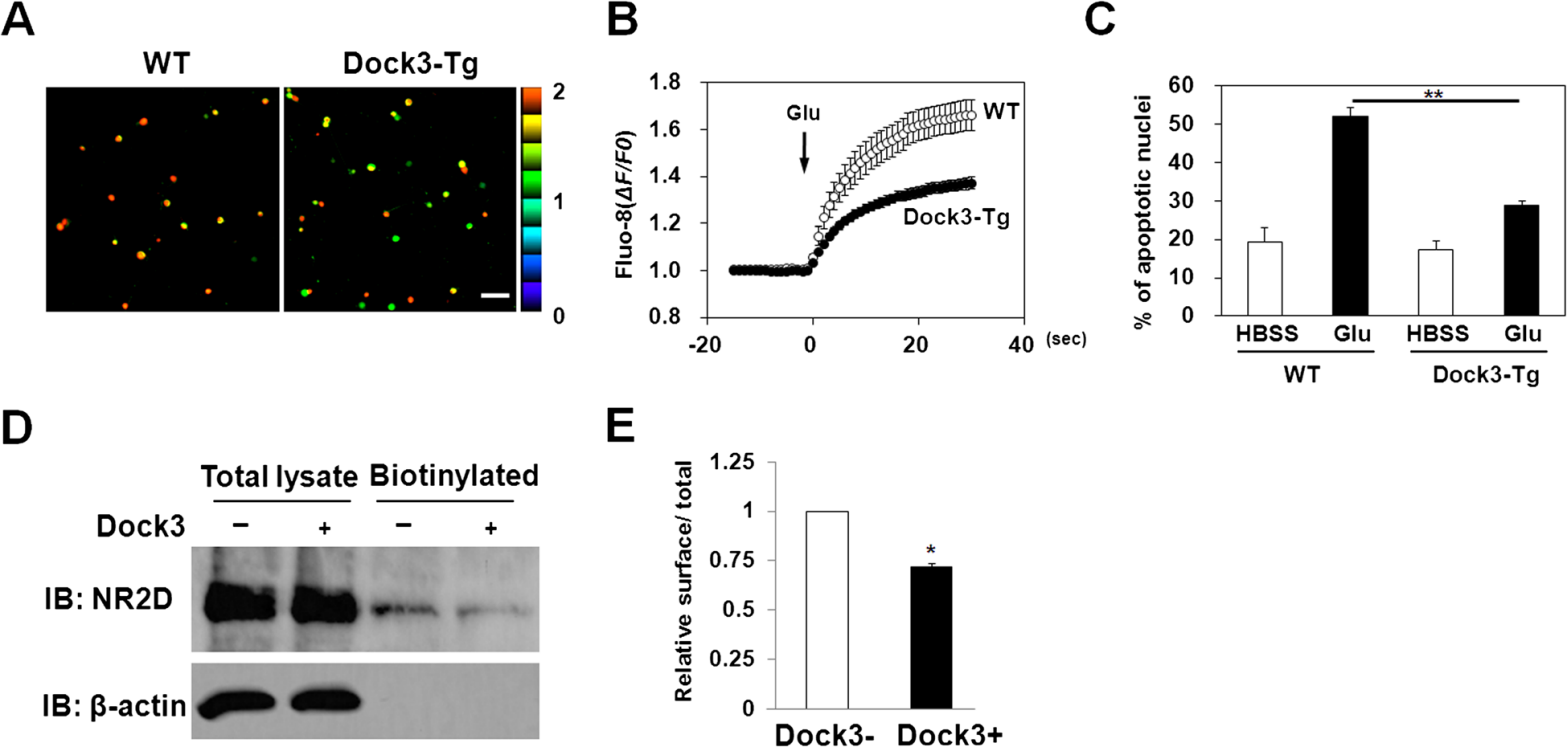
**Overexpression of Dock3 inhibits glutamate-induced intracellular Ca**^**2+**^**elevation and prevents glutamate-induced apoptosis in RGCs.** (**A**) RGCs from WT or Dock3-transgenic (Tg) mice were labeled with Fluo-8 acetoxymethyl ester for 30 min, and then 300 μM glutamate (Glu) was added. Fluorescence-ratio images are displayed in pseudocolor, as indicated by the color bar on the right side of the images. Pseudocoloring represents ratios between 0 and 2, corresponding to 1, which is defined as the basal fluorescence intensities before Glu stimulation. Left and right panels show ratio images for WT and Dock3-Tg, respectively. Scale bar: 100 μm. (**B**) Changes in Fluo-8 fluorescence are expressed as ΔF/F0, where F0 is the basal fluorescence intensity before Glu stimulation. Glu was added as indicated. Data represent the mean ± SE from eight independent experiments. (**C**) Fragmented or shrunken nuclei were detected by Hoechst staining 24 hr after control (HBSS) or glutamate (Glu) treatment. **p < 0.0001 for WT Glu vs. Dock3-Tg Glu. WT, wild-type mice; Dock3-Tg, Dock3 transgenic mice. (**D**) N2A cells were transfected with NR1 and NR2D with (Dock3+) or without Dock3 (Dock3-). Surface proteins were biotinylated using sulfo-NHS-SS-biotin, immunoprecipitated with streptavidin beads and probed with NR2D antibodies. Data are from a single experiment, which is representative of five experiments that yielded similar results. (**E**) Relative ratio of biotinylated protein to total protein. *p < 0.05. Data represent the mean ± SE from five independent experiments.

Next, we examined the functional consequences of the interaction between NR2D and Dock3, using primary cultures of RGCs. Previously, we reported that glutamate-induced Ca^2+^ elevation and apoptosis in RGCs are mediated by NMDARs [22]. Therefore, we examined the effects of overexpressing Dock3 on glutamate-induced Ca^2+^ elevation and apoptosis in cultured RGCs. Fluorescence-ratio images, displayed in pseudocolor in Figure 6A, demonstrated that glutamate markedly increased the intracellular Ca^2+^ concentration in RGCs from WT mice. Overexpression of Dock3 significantly inhibited glutamate-induced intracellular Ca^2+^ elevation in RGCs (Figure 6A, B). Because an increase in intracellular Ca^2+^ in RGCs mediated by NMDARs is a key step in initiating apoptosis [22,23], we next examined the neuroprotective effect of Dock3 on RGCs. Whereas 300 μM glutamate induced apoptosis in RGCs from WT mice (Figure 6C), overexpression of Dock3 significantly inhibited glutamate-induced apoptosis (Figure 6C). Together, these findings demonstrate that overexpression of Dock3 inhibits glutamate-induced intracellular Ca^2+^ elevation and prevents glutamate-induced apoptosis in RGCs, suggesting that the interaction between NR2D and Dock3 suppresses the function of NR2D-containing NMDARs.

### Dock3 suppresses the surface expression of NR2D

Because NMDARs undergo regulated endocytosis [24-26], overexpression of Dock3 may reduce glutamate-induced Ca^2+^ elevation and apoptosis in RGCs by reducing the number of NMDARs on the cell surface. To investigate whether Dock3 suppresses the surface expression of NR2D, we used N2A neuroblastoma cells. N2A cells were transfected with expression plasmids encoding NR1 and NR2D, with or without a plasmid harboring the full-length Dock3 cDNA. Cell-surface receptors were then quantified using a biotinylation assay. As shown in Figure 6D and E, the number of biotinylated NR2D subunits significantly decreased upon co-expression of Dock3, however, no changes was observed in the total amount of NR2D. These results suggest that Dock3 suppresses the surface expression of NR2D-containing NMDARs, thereby inhibiting glutamate-induced Ca^2+^ elevation and apoptosis in RGCs.

## Discussion

In the present study, we show that NR2D is involved in the progressive loss of RGCs in GLAST-deficient mice, an animal model of NTG. In addition, we identify Dock3 as a novel NR2D-interacting protein and show that the interaction between NR2D and Dock3 protects RGCs from excitotxicity by reducing the surface expression of NR2D.

This is the first direct *in vivo* evidence of NR2D-NMDAR-mediated excitotoxicity in the context of glaucoma. In *GLAST*^*−/−*^ mice, NR2D deficiency only partially prevented loss of RGCs, whereas inhibition of NMDARs with memantine almost completely prevented RGC loss [16]. This may be due to the involvement of other NR2 subunits in RGC degeneration, especially the NR2C subunit, given that NR2C-containing NMDARs are expressed in RGCs [15] and are the targets of therapeutic memantine activity [11]. NR2D deficiency prevented NMDA-induced-excitotoxic retinal cell death specifically in GCL, but not in INL. This can be explained by the lower expression level of NR2D in neurons of INL compared with those in GCL and by the contribution of other NR2 subunits to NMDA-induced cell death in INL.

Considering the high frequency of glaucoma in Alzheimer’s disease patients [27], common mechanisms such as NR2D-NMDAR-mediated excitotoxicity might contribute to both diseases. In addition, NR2D mediates the capacity of PCP to induce locomotor hyperactivity in a novel environment, behavior that is thought to model positive symptoms in schizophrenia [12]. The genomic region that contains the NR2D gene locus has also been suggested to contribute to susceptibility to schizophrenia in a Japanese population [28]. NR2D is expressed in the adult mammalian brain, including the hippocampus, cortex, thalamus, and retina, all of which are CNS regions thought to be involved in Alzheimer’s disease, schizophrenia, and glaucoma. Taken together, these findings suggest that NR2D may play roles in various neuropsychiatric diseases, including glaucoma, Alzheimer’s disease, and schizophrenia, and may be a target for the development of novel drugs for the treatment of these neuropsychiatric diseases.

Multiple studies show that protein-protein interactions occurring at the intracellular C-terminal domains of NMDARs control the function and localization of these receptors. Whereas numerous proteins that interact with NR2A and NR2B have been identified [20,29,30], few directly interact with the NR2D subunit. The Abl tyrosine kinase is reported to interact directly with NR2D, but this interaction has no direct effect on NR2D function [31].

In the present study, we identified Dock3 as a novel NR2D-interacting protein. Dock3 was first identified as a presenilin (PS)-binding protein (PBP) [21] expressed in neurons and the testis, and is involved in cell adhesion and neurite outgrowth [32]. Dock3 may be linked to the pathogenesis of Alzheimer’s disease: it is absent from the soluble fraction of samples taken from Alzheimer’s disease brains [21], accumulates in neurofibrillary tangles [33], and regulates Aβ secretion [34]. Consistent with this hypothesis, deletion of Dock3 results in axonal degeneration and sensorimotor impairments [35]. Previously, we showed that overexpression of Dock3 induces optic nerve regeneration following injury [36]. In addition, we showed the role of Dock3 in glutamate-induced Ca^2+^ elevation and apoptosis of RGCs. In primary cultured RGCs, NMDARs contribute to Ca^2+^ elevation and apoptosis induced by 300 μM glutamate [22]. Overexpression of Dock3 significantly inhibited these glutamate-induced responses. Considering the direct interaction between Dock3 and NR2D, reflected in their co-localization, and the role of NR2D in NMDA-induced retinal cell death, these results suggest that Dock3 may play an important role in protecting RGCs from excitotoxicity through modulation of NR2D function.

Further mechanistic insights into the inhibition of glutamate-induced Ca^2+^ elevation and apoptosis of RGCs by overexpression of Dock3 were gained by conducting biochemical analyses of the surface expression of NR2D. Co-expression of Dock3 suppressed the expression of NR2D on the surface of the plasma membrane. Although further studies are required to clarify the mechanism by which Dock3 expression reduces the surface expression of NR2D, we can suggest two possible explanations. The first is that Dock3 could activate Rac1; activated Rac1 may then reduce the surface expression of NR2D by promoting endocytosis, similar to Kir2.1 channels [37]. The second possible explanation is that Dock3 interacts with both Fyn [36] and NR2D; Fyn may then regulate the surface expression of NR2D through tyrosine phosphorylation. The NR2D protein is developmentally regulated by tyrosine phosphorylation *in vivo*, suggesting that tyrosine phosphorylation may be important for regulating the functions of this NMDAR subunit in the mammalian CNS [38].

## Conclusions

We show here that NR2D is involved in excitotoxic degeneration of retinal cells and that Dock3 is a novel NR2D-interacting protein. Moreover, the interaction between NR2D and Dock3 protects RGCs from excitotoxicity by reducing the surface expression of NR2D. Identification of chemical compounds that can increase the expression of Dock3, or otherwise reduce the surface expression of NR2D, might have therapeutic benefit for the treatment of neurodegenerative disorders such as Alzheimer’s disease and glaucoma.

## Methods

### Animals

GLAST and NR2D mutant mice were described previously [39,40]. Double-knockout mice (*GLAST*^*−/−*^*/NR2D*^*−/−*^) and homozygous GLAST-knockout mice (*GLAST*^*−/−*^) were obtained by crossing double heterozygous mice (*GLAST*^*+/−*^*/NR2D*^*+/−*^). The homozygous NR2D-knockout mice (*NR2D*^*−/−*^) were obtained by crossing heterozygous mice (*NR2D*^*+/−*^). The genotypes of the mutant mice were determined as described previously [39,40]. Dock3 transgenic mice overexpress wild-type Dock3 under the control of the actin promoter [36]. In all experiments, age-matched WT and *GLAST*^*−/−*^ littermate controls were used. All mice were of the C57BL/6 J genetic background, and all animal procedures were approved by the Animal Committee of Tokyo Medical and Dental University (0130166C).

### Histological analysis

Mice were deeply anesthetized by diethyl ether. Eyes from mice at postnatal day 35 (P35) were enucleated, fixed in Davidson’s solution fixative [41] overnight at 4°C, and dehydrated in 70% ethanol for three days at 4°C. The fixed eyes were then embedded in paraffin wax. Sections (7-μm thickness) of embedded retinal specimens were cut and stained with hematoxylin and eosin. The number of neurons in the GCL was counted from one ora serrate, through the optic nerve, to the other ora serrata. The average number of neurons in the GCL per eye was calculated from three sections of each retina.

### Intravitreal injection of NMDA

WT and *NR2D*^*−/−*^ mice (5 weeks old) were anesthetized by intraperitoneal injection of 50 mg/kg sodium pentobarbital, and their pupils were dilated with tropicamide. A single 2-μl injection of 20 mM NMDA in 0.1 M PBS (pH 7.40) was administered intravitreally into the right eye of each mouse, thereby delivering a dose of 40 nmol of NMDA. The same volume of PBS was administered to the contralateral (left) eye as control. To avoid lens injury, injections were performed under a stereomicroscope through a 32-gauge needle (Dentronics) connected to a 10-μl Hamilton syringe (Hamilton); the needle was inserted approximately 1 mm behind the corneal limbus.

### TUNEL assay

Twenty-four hours after the NMDA or PBS injection, eyes were enucleated, fixed in Davidson fixative overnight at 4°C, embedded in paraffin, and sectioned (5-μm thickness). Apoptotic cells were labeled using the DeadEnd Fluorometric TUNEL (terminal deoxynucleotidyl transferase dUTP nick end labeling) System (Promega) according to the manufacturer's instructions. TUNEL-positive cells in GCL and INL were counted manually under a microscope. The average number of TUNEL-positive cells per eye was calculated for three sections of each retina.

### Yeast two-hybrid screening

Yeast two-hybrid screening was performed by Hybrigenics Services, S.A.S., Paris, France. The coding sequence for the cytoplasmic region (aa 895–1323) of mouse NR2D (GenBank accession number gi: 144922605) was PCR-amplified and cloned into pB27 as a C-terminal fusion to LexA. The resulting plasmid, pB27 (N-LexA-NR2D-C), was used as a bait to screen a random-primed mouse adult brain cDNA library cloned into vector pP6. The bait strain/prey strain mating was spread on a medium lacking tryptophan, leucine, and histidine, and supplemented with 5 mM 3-aminotriazole to overcome bait auto-activation. A total of 357 positive clones were obtained out of 82 million interactions tested. The prey fragments of the positive clones were amplified by PCR and sequenced at their 5’ and 3’ junctions. The resulting sequences were used to identify the corresponding interacting proteins in the GenBank database (NCBI).

### DNA constructs

Full-length mouse NR1 and NR2D cDNAs were amplified from vectors described previously [42,43] and subcloned into vector pEGFP C1 (Clontech), such that the NR2D protein was fused in frame with the C-terminal EGFP epitope. A similar plasmid was constructed for expression of the region containing the NR2D CT (aa 895–1323). The plasmid encoding mouse Dock3 cDNA (GenBank accession number gi: 148277095) was described previously [36]. The selected interacting domain (SID) of Dock3 was amplified by PCR and subcloned into vector pEF1/myc-His A (Invitrogen) such that the Dock3 protein was fused in frame with the N-terminal myc and His epitopes. All expression plasmids were confirmed by DNA sequencing.

### Cell culture and transfection

HEK 293 T cells were cultured on 10-cm plates in Dulbecco’s modified Eagle’s medium (Sigma), supplemented with 10% fetal bovine serum (GIBCO) at 37°C in atmosphere containing 5% CO_2_. When the cells reached 70% confluence, they were transiently transfected with cDNA constructs (up to 6 μg total) using the GeneJuice transfection reagent (Novagen).

### Immunoprecipitation and immunoblotting

Cells were harvested 48 h after transfection and resuspended in cold lysis buffer (50 mM Tris–HCl, 1% Nonidet P-40, 5 mM EDTA, 150 mM NaCl, 0.5% Na-deoxycholate, 1 mM MgCl_2_, 1 mM DTT, 1 mM Na_3_VO_4_, 1 mM NaF, 1 mM phenylmethylsulfonyl fluoride (PMSF), and Complete Protease Inhibitor Cocktail [Roche]). Samples were left on ice for 30 min and sonicated briefly. The insoluble fraction was removed by centrifugation at 15,000 rpm for 15 min. Protein concentration was determined using BCA Protein Assay kit (Sigma-Aldrich). Total cell lysates (TCLs) were boiled in the presence of 2× sample buffer. Immunoprecipitation was performed with 50 μl of anti-Myc magnetic beads (clone PL14; MBL), anti-GFP magnetic beads (clone RQ2; MBL), and 400 μg of cell homogenate according to the manufacturer’s protocol. Immunoprecipitates and TCL were separated by SDS-polyacrylamide gel electrophoresis (SDS-PAGE) and transferred onto polyvinylidene difluoride (PVDF, Millipore) membranes. The membranes were blocked with 10% skim milk/PBST (PBS containing 0.05% Tween 20) solution for 1 h at room temperature, and then treated with primary antibodies. The following antibodies were used for the blots: anti–Myc-tag rabbit polyclonal antibody (1:1000; MBL) and anti-GFP rabbit polyclonal Living Colors Av peptide antibody (1:100; Clontech Laboratories). After 1 h at room temperature, the membrane was washed three times in PBST for 30 min and incubated for 1 h in horseradish peroxidase (HRP)-conjugated second antibody (1:10,000; Jackson ImmunoResearch Laboratories, Inc.).

For *in vivo* immunoprecipitation, 90 mg of brain tissue was collected from mice at embryonic day 18 and homogenized using a POLYTRON PT 1200E homogenizer (Kinematica AG) in 500 ul cold lysis buffer (300 mM NaCl, 5 mM Tris-Cl [pH 7.5], 0.5% Triton X-100, and Complete Protease Inhibitor Cocktail). Co-immunoprecipitation was performed according to the method previously described [44] with some modifications. Briefly, the sample was left on ice for 1 h, spun at 10,000 *g* for 20 min at 4°C, and the supernatant was collected, and the protein concentration was determined using BCA Protein Assay kit. NR2D antibody [45] (1 μg) and Dock3 antibody [46] (0.5 μg) were added to 500 μl of pre-cleared lysate and incubated at 4°C overnight. Protein G Sepharose (50 μl) was then added to collect the immunoprecipitate. Samples were incubated at 4°C for 2 h and centrifuged at 1,000 *g* for 1 min, and supernatant was removed. The precipitate was washed three times with 1 ml of lysis buffer. Each sample was diluted with 50 μl of 2× sample buffer and run on an SDS polyacrylamide gel (7.5%); an equal volume (20 μl) of each sample was loaded onto the gel. Separated proteins were transferred to PVDF membranes. The membranes were then incubated with an anti-NR2D guinea-pig polyclonal antibody (1:1000) and an anti-Dock3 rabbit polyclonal antibody (1:1000) at 4°C overnight. The membrane was washed three times in TBST (TBS containing 0.05% Tween 20) for 30 min and incubated for 1 h with an HRP-conjugated secondary antibody (1:5,000–1:10,000). SuperSignal West Femto Maximum Sensitivity Substrate (Thermo Scientific) was used to visualize the immunoreactive proteins.

### Immunohistochemistry

Mice were deeply anesthetized by diethyl ether and perfused with 0.1 M PBS and then with 4% PFA in 0.1 M PB. Eyes were immediately enucleated and immersed in the same fixative for 2 hours at 4°C, followed by immersion in a sucrose solution (30% in PB) overnight at 4°C. Eyes were embedded in OCT compound (Sakura Finetechnical Co. Ltd) and frozen on dry ice. The eyes were sectioned (10-μm thickness) using a cryostat. After washing in PBS, the sections were blocked with 5% normal horse serum diluted in PBS with 0.2% Triton X-100 for 30 minutes at room temperature. Sections were incubated overnight at 4°C with a goat polyclonal antibody against NR2D (1:50; Santa Cruz Biotechnology, INC) and a rabbit polyclonal antibody against Dock3 (1:100). Sections were then incubated with secondary antibodies against goat and rabbit IgG (Alexa Furo 488, 1:1000; Alexa Furo 568, 1:500; both from Molecular Probes) for 1 h at room temperature. After a final rinse in PBS, the sections were cover-slipped with Fluoromount (Diagnostic BioSystems). Images were recorded with an LSM-510 META confocal laser microscope (Carl Zeiss).

### Primary culture of mouse retinal ganglion cells

C57BL/6 J or Dock3-Tg mice (7–10 days old) were used for primary culture of RGCs according to Winzeler *et al.*[47] with some modifications. Briefly, retinae were digested with papain (16.5 units/ml) for 45 min at 37°C, triturated in Minimum Essential Medium (MEM; Invitrogen) containing 0.15% trypsin inhibitor (Roche Applied Science), and then triturated again in MEM containing 1% trypsin inhibitor. The cell suspension was incubated on a first panning plate (150 mm Petri dish) coated with Bandeiraea lectin I (L-1100; Vector Laboratories, Inc.) for 10 min at room temperature. Non-adherent cells were incubated for 45 min on a second panning plate (100 mm Petri dish) coated with goat anti-mouse IgG + IgM (H + L) (Jackson ImmunoResearch Laboratories, Inc.) and mouse anti-mouse Thy1.2 IgM antibodies (MCA02R; AbD Serotec, Oxford, UK). The panning plate was washed with PBS, and adherent RGCs were released by treatment with 0.125% trypsin for 10 min at 37°C. The RGC suspension was mixed with 30% fetal bovine serum and centrifuged at 200 *g* for 10 min. RGCs were suspended in medium containing 1 mM glutamine, 5 μg/ml insulin, 60 μg/ml N-acetylcysteine, 62 ng/ml progesterone, 16 μg/ml putrescine, 40 ng/ml sodium selenite, 0.1 mg/ml BSA, 40 ng/ml triiodothyronine, 0.1 mg/ml transferrin, 1 mM sodium pyruvate, 2% B27 supplement (Invitrogen), 10 μM forskolin (Sigma), 50 ng/ml brain-derived neurotrophic factor (BDNF; PeproTech, Rocky Hill, NJ), 50 ng/ml ciliary neurotrophic factor (CNTF; PeproTech), and 50 ng/ml basic fibroblast growth factor (bFGF; PeproTech) in Neurobasal medium (Invitrogen). Ninety-six–well culture plates were coated with poly-D-lysine (Sigma) and laminin (Sigma) and mouse RGCs were plated at a density of 4,000 cells/well (or 4,000 cells/culture insert for μ-dishes (ibidi)) and cultured for at least 10 days before the experiments.

### Induction and detection of apoptosis induced by glutamate

RGCs were washed twice (15-min incubation, ×2) with Hank’s Balanced Salt Solution (HBSS; Invitrogen) containing 2.4 mM CaCl_2_ and 20 mM HEPES without magnesium. Subsequently, RGCs were incubated for 2 h at 37°C with or without 300 μM glutamate and 10 μM glycine (a co-activator of NMDARs) in HBSS containing 2.4 mM CaCl_2_ and 20 mM HEPES without magnesium. After HBSS or glutamate treatment, RGCs were cultured for 22 h at 37°C in the same medium to culture the RGCs, but without forskolin, BDNF, CNTF, and bFGF. To detect apoptosis using Hoechst 33342 (Dojindo), RGCs were washed once with PBS and incubated with 1 μg/ml Hoechst 33342 for 15 min at room temperature. Fluorescent images were randomly taken (four images/well) using an Olympus IX71 fluorescence microscope. For each treatment, at least eight images were taken from two wells of a 96-well plate. Fragmented or shrunken nuclei stained with Hoechst dye were deemed apoptotic, and neurons with round and smooth nuclei were counted as healthy. More than 200 neurons for each treatment were counted by a researcher blinded to the identity of the samples.

### Measurement of intracellular calcium

Mouse RGCs were incubated for 30 min at 37°C in culture medium with 3 μM Fluo-8 acetoxymethyl ester (AAT Bioquest). Cells were washed twice (15-min incubation, ×2) with HBSS containing 2.4 mM CaCl_2_ and 20 mM HEPES without magnesium, then stimulated with 300 μM glutamate and 10 μM glycine. Fluorescence images were acquired every 500 msec using an ORCA-R2 digital CCD camera (Hamamatsu Photonics) and analyzed using the MetaFluor fluorescence-ratio imaging software (Molecular Devices).

### Surface-biotinylation assay

Neuro 2A cells were plated at a density of 2 × 10^5^/well in 6-well plates and cultured in 95% air/5% CO_2_ at 37°C. The cells were transiently co-transfected with the cDNAs encoding NR1 and NR2D with (Dock3+) or without (Dock3-) Dock3. Forty-eight hours after transfection, cells were incubated in PBS containing 1.5 mg/ml Sulfo-NHS-SS-biotin (Pierce) for 20 min at 4°C. Surface biotinylation was stopped by removing that solution and incubating the cells in 10 mM ice-cold glycine in PBS for 20 min. Cells were rinsed twice in PBS and then lysed in 200 μl PBS with Complete Protease Inhibitor Cocktail, 0.1% SDS, and 1% Triton X-100. A fraction (15%, 30 μl) of the cell lysate was removed to measure total protein concentration and for total input; the remaining 85% (170 μl) of the cell lysate was incubated with 70 μl of 50% avidin-agarose (Sigma) overnight at 4°C. After washing three times with lysis buffer, bound proteins were resuspended in 30 μl of 2× sample buffer and boiled. Samples were analyzed by SDS-PAGE followed by Western blotting using anti-NR2D guinea-pig polyclonal antibody (1:1000). The data were quantified by measuring the ratios between intensities of the biotinylated and total NR2D bands using the Image Lab software (Bio-Rad). Surface/total ratios from the Dock3- control were assigned a value of 1. Ratios of the Dock3+ groups were expressed relative to the controls and averaged.

### Statistical analysis

All data are expressed as mean ± S.E. Statistical analyses were conducted using Student's *t*-test for comparison between two samples, or one-way ANOVA followed by Bonferroni’s test for multiple comparisons, using the SPSS 17.0 software package. *P* values < 0.05 were considered statistically significant.

## Abbreviations

CNS: Central nervous system; DHR-1: Dock homology region 1; DHR-2: Dock homology region 2; Dock3: Dedicator of cytokinesis 3; GCL: Ganglion cell layer; GLAST: Glutamate aspartate transporter; HEK: Human embryonic kidney; INL: Inner nuclear layer; MOCA: Modifier of cell adhesion protein; NMDAR: N-methyl-D-aspartate receptor; NTG: Normal tension glaucoma; PBP: Presenilin binding protein; PBS: Phosphate-buffered saline; PCP: Phenycyclidine; PMSF: Phenylmethylsulfonyl fluoride; PS: Presenilin; PVDF: Polyvinylidene difluoride; RGC: Retinal ganglion cell; TCL: Total cell lysate; WT: Wild-type

## Competing interests

The authors declare that they have no competing interests.

## Authors’ contributions

KT, TA and NB conceived and designed the experiments. NB carried out all experiments except the experiments performed on primary cultured RGCs and analyzed the data. HH carried out the experiments performed on primary cultured RGCs and analyzed the data. KN, TH and MM contributed reagents and materials. KT and NB wrote the paper. All authors have read and approved the manuscript for publication.
